# Thiostrepton as a Potential Therapeutic Agent for Hepatocellular Carcinoma

**DOI:** 10.3390/ijms25179717

**Published:** 2024-09-08

**Authors:** Guifeng Su, Qianqing Yang, Heyang Zhou, Ying Huang, Shiyun Nie, Dan Wang, Guangchao Ma, Shaohua Zhang, Lingmei Kong, Chenggang Zou, Yan Li

**Affiliations:** 1 Key Laboratory of Medicinal Chemistry for Natural Resource, Ministry of Education; Yunnan Key Laboratory of Research and Development for Natural Products, School of Pharmacy, Yunnan University, Kunming 650500, China; suguifeng@mail.kib.ac.cn (G.S.); yangqianqing@mail.ynu.edu.cn (Q.Y.); hyzhou@mail.ynu.edu.cn (H.Z.); huangying@mail.yun.edu.cn (Y.H.); nieshiyun@mail.ynu.edu.cn (S.N.); wangdan@mail.ynu.edu.cn (D.W.); guangchaoma@mail.ynu.edu.cn (G.M.); shzhang_cpu@126.com (S.Z.); konglingmei@ynu.edu.cn (L.K.); 2 State Key Laboratory for Conservation and Utilization of Bio-Resources in Yunnan, School of Life Sciences, Yunnan University, Kunming 650500, China; 3 State Key Laboratory of Phytochemistry and Plant Resources in West China, Kunming Institute of Botany, Chinese Academy of Sciences, Kunming 650201, China; 4 University of Chinese Academy of Sciences, Beijing 100049, China

**Keywords:** thiostrepton, hepatocellular carcinoma, mitochondrial impairment, ROS, mitophagy

## Abstract

Due to limited drug efficacy and drug resistance, it is urgent to explore effective anti-liver cancer drugs. Repurposing drugs is an efficient strategy, with advantages including reduced costs, shortened development cycles, and assured safety. In this study, we adopted a synergistic approach combining computational and experimental methods and identified the antibacterial drug thiostrepton (TST) as a candidate for an anti-liver cancer drug. Although the anti-tumor capabilities of TST have been reported, its role and underlying mechanisms in hepatocellular carcinoma (HCC) remain unclear. TST was found here to inhibit the proliferation of HCC cells effectively, arresting the cell cycle and inducing cell apoptosis, as well as suppressing the cell migration. Further, our findings revealed that TST induced mitochondrial impairment, which was demonstrated by destroyed mitochondrial structures, reduced mitochondria, and decreased mitochondrial membrane potential (MMP). TST caused the production of reactive oxygen species (ROS), and the mitochondrial impairment and proliferation inhibition of HCC cells were completely restored by the ROS scavenger N-acetyl-L-cysteine (NAC). Moreover, we discovered that TST induced mitophagy, and autophagy inhibition effectively promoted the anti-cancer effects of TST on HCC cells. In conclusion, our study suggests TST as a promising candidate for the treatment of liver cancers, and these findings provide theoretical support for the further development and potential application of TST in clinical liver cancer therapy.

## 1. Introduction

Liver cancer, a prevalent malignance in the digestive system with the third-highest mortality rate, often develops following hepatitis, liver fibrosis, and cirrhosis [1,2]. Primary liver cancer encompasses HCC, hepatoblastoma, intrahepatic cholangiocarcinoma (ICC), and mixed HCC and ICC [3]. HCC accounts for approximately 80% of PLC cases, making it a major focus [1]. The main treatments for HCC currently involve surgical resection, liver transplantation, and chemotherapy [4]. Nevertheless, the asymptomatic nature of HCC in its early stages, coupled with its propensity for recurrence and drug resistance in the intermediate and advanced stages, has resulted in unsatisfactory outcomes of these treatments [4,5,6]. Hence, there is an urgent need to develop promising new drugs for the effective treatment of HCC.

Drug repurposing, also known as repositioning, represents a promising direction for the development of potential drugs [7]. This approach offers several advantages compared to developing a new drug from the very beginning, such as cost savings, shortened development cycles, and assured safety [8,9,10]. Current drug repurposing efforts have yielded promising outcomes for anti-cancer drugs, among which thalidomide is particularly notable [11]. Drug repurposing has often depended on serendipity or retrospective clinical trial observations, but the introduction of bioinformatics has brought about a substantial improvement in research methods, making them more systematic, dependable, and efficient in terms of cost and time [12,13].

As the energy plant of cells, mitochondria impairment has emerged as a potential cancer therapy strategy to overcome tumor growth by disrupting the MMP, which results in the release of cytochrome c and subsequent cleavage of caspases, eventually triggering apoptosis. However, damaged mitochondria can be degraded by mitophagy, the selective autophagy of mitochondria [14]. Current research suggests that defects in mitophagy lead to the accumulation of abnormal mitochondria, resulting in altered cellular metabolism and cell fate determination and thereby promoting the occurrence and development of tumors [15]. Under chemotherapy, certain tumor cells develop drug resistance through mitophagy, thereby promoting cell survival [16,17,18]. Therefore, thorough research on mitophagy holds the promise of offering new insights for the development of clinical anti-cancer treatment strategies.

TST, originally an antibacterial drug, has increasingly been discovered to have anti-tumor activity in cancers such as osteosarcoma [19], gastric cancer [20], and breast cancer [21], but the anti-liver cancer activity of TST has not been reported and the underlying mechanisms remain unknown. In the present study, we utilized a bioinformatic approach to screen drug candidates from the PRISM repurposing dataset [22] and identified TST as a potential candidate for the treatment of liver cancers. We found that TST induced cell cycle arrest and apoptosis and inhibited migration in HCC cells, thereby exerting its anti-liver cancer activity. Upon further investigation, we unveiled that the anti-tumor activity of TST was attributed to mitochondrial impairment which was mediated by ROS induction in HCC cells. Furthermore, TST induced mitophagy in liver cancer cells, and mitophagy inhibition enhanced the anti-liver cancer effects of TST. Taken together, these findings provide informative data and theoretical support for the further clinical development and application of TST as an anti-liver cancer agent.

## 2. Results

### 2.1. TST Is Identified as a Potential Agent against HCC

**Figure 1:**
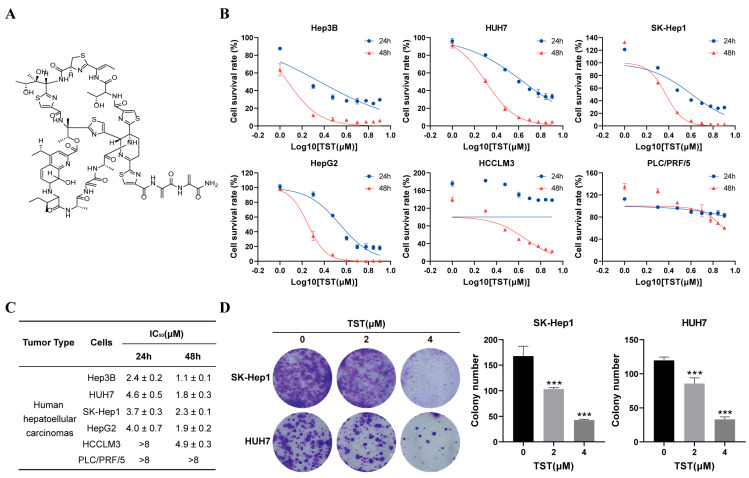
TST suppresses the proliferation of HCC. (**A**) Chemical structure of TST. (**B**) The cellular viability of HCC cells incubated with various concentrations of TST for 24 or 48 h was assessed using MTS solution. (**C**) IC_50_ calculated from dose–response curves. (**D**) Colony formation in SK-Hep1 and HUH7 cells following 2 weeks of TST treatment. Quantification of cell colony numbers is shown on the right. *** *p* < 0.001 versus the control group.

To explore potential drugs for the treatment of liver cancer, we created a drug analysis program and performed a screening of the PRISM database [22] with the flowchart shown in Figure S1A. TST was found to be a promising anti-liver cancer candidate, which has not been reported (Figure S1B,C). The proliferation inhibitory activity of TST on HCCs was verified in Hep3B, HUH7, SK-Hep1, HepG2, HCCLM3, and PLC/PRF/5 cells. All the cells were treated with TST for 24 or 48 h (Figure 1A) and subjected to MTS assay, and the cell viability data revealed a dose- and time-dependent decrease in the survival of HCC cells upon TST treatment, with the IC_50_ values listed (Figure 1B,C). The IC_50_ results indicated that TST exhibits strong activity against Hep3B, SK-Hep1, HUH7, and HepG2 cells.

With SK-Hep1 recognized for its high invasiveness and HUH7 characterized by a strong proliferative ability, these two highly malignant cell lines were selected for subsequent experiments. Consistently, the number of colonies decreased in SK-Hep1 and HUH7 cells challenged with TST in the colony formation assay, further confirming the substantial inhibitory effect of TST on the HCC cells (Figure 1D).

### 2.2. TST Inhibits the Migration of HCC Cells

**Figure 2:**
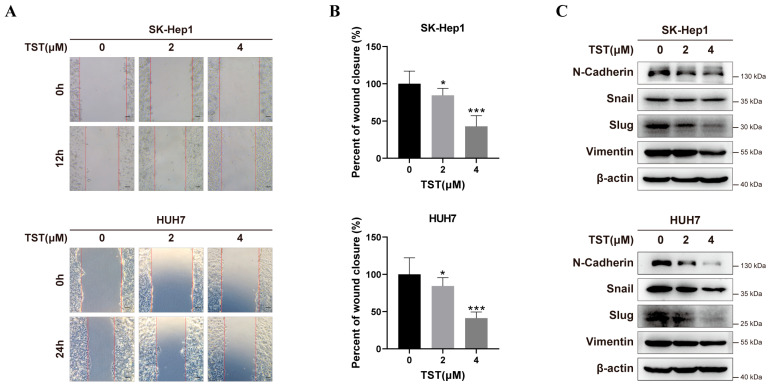
TST suppresses the migration of HCC cells. (**A**) The migratory capacity of liver cancer cells treated with TST was assessed with cell scratch assay. Scale bar: 100 μm. (**B**) Statistical analysis of cell migration rate was displayed in histograms. * *p* < 0.05, and *** *p* < 0.001 versus the control group. (**C**) Western blot analysis revealed the expression levels of migration-related proteins, including N-cadherin, Snail, Slug, and Vimentin, in HCC cells subjected to TST treatment.

HCC is a highly malignant tumor with frequent metastasis, which is associated with a poor prognosis. Thus, the efficacy of TST against metastasis was investigated. We observed a dose-dependent inhibition of the migration of HCC cells with TST incubation (Figure 2A,B). Additionally, migration-related proteins, including N-cadherin, Snail, Slug, and Vimentin, were significantly decreased in HCC cells with TST treatment (Figure 2C). Overall, these results indicated that TST inhibited the migration of HCC cells.

### 2.3. TST Arrests the Cell Cycle and Induces Cell Apoptosis of HCC Cells

**Figure 3:**
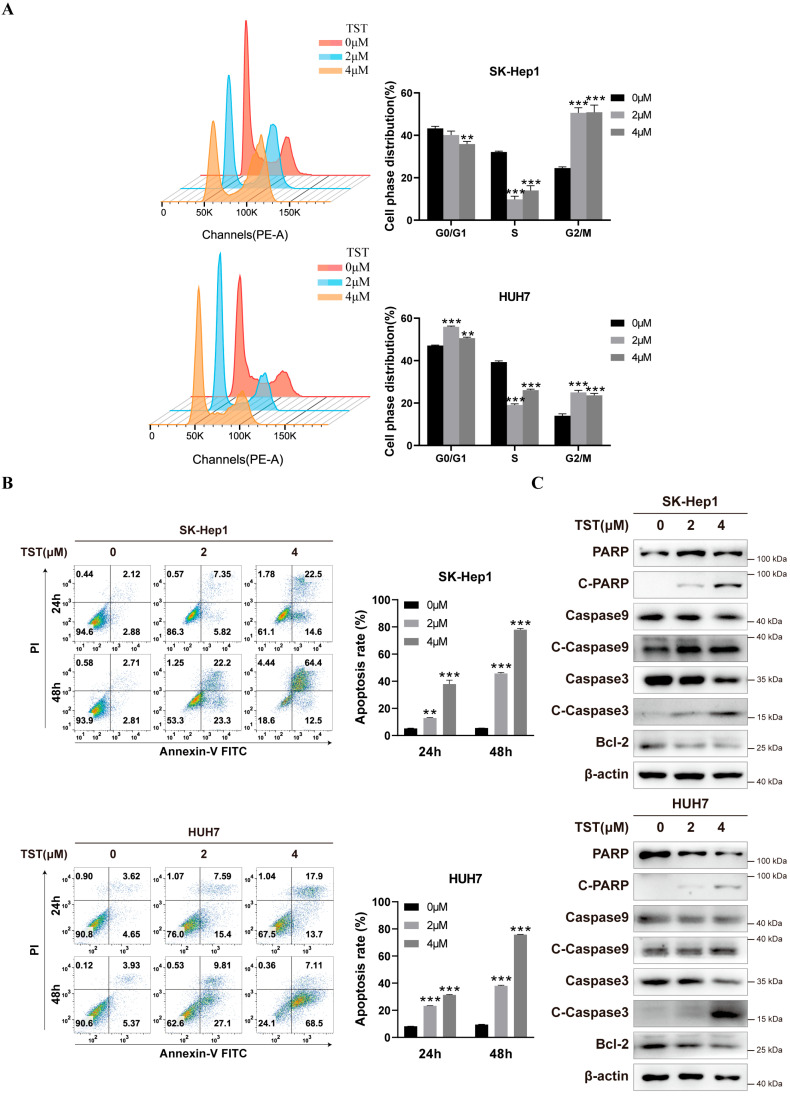
TST induces apoptosis and arrests the cell cycle in HCC cells. (**A**) The cell phase distribution of HCC cells after 24 h of TST treatment, with the analysis results presented as histograms. ** *p* < 0.01, and *** *p* < 0.001 versus the control group. (**B**) Apoptosis of HCC cells treated with TST was assessed with flow cytometry. Statistical analysis of cell apoptosis rates was displayed in histograms. ** *p* < 0.01 and *** *p* < 0.001 versus the control group. (**C**) Apoptosis-related proteins were detected by western blotting using proteins extracted from liver cancer cells treated with TST for 24 h.

The effect of TST on cell cycle distribution was assessed with propidium iodide (PI) staining and flow cytometry. Cells were incubated with TST for 24 h and subjected to cell cycle analysis, and the percentage of cells in the G2/M phase increased compared to the control group, indicating TST-induced G2/M cell cycle arrest in HCC cells (Figure 3A). Next, cell apoptosis was analyzed and the results revealed an obvious increase in apoptotic HCC cells treated with TST in both a time- and dose-dependent manner (Figure 3B). Furthermore, apoptosis marker proteins, including cleaved caspase-3, cleaved caspase-9, and cleaved poly-(ADP-ribose) polymerase 1 (PARP), showed a concentration-dependent increase after TST treatment with the anti-apoptotic protein Bcl-2 downregulated (Figure 3C). Taken together, these results demonstrated that TST induced G2/M cell cycle arrest and cell apoptosis of HCCs.

### 2.4. TST Impairs the Mitochondria in HCC Cells

**Figure 4:**
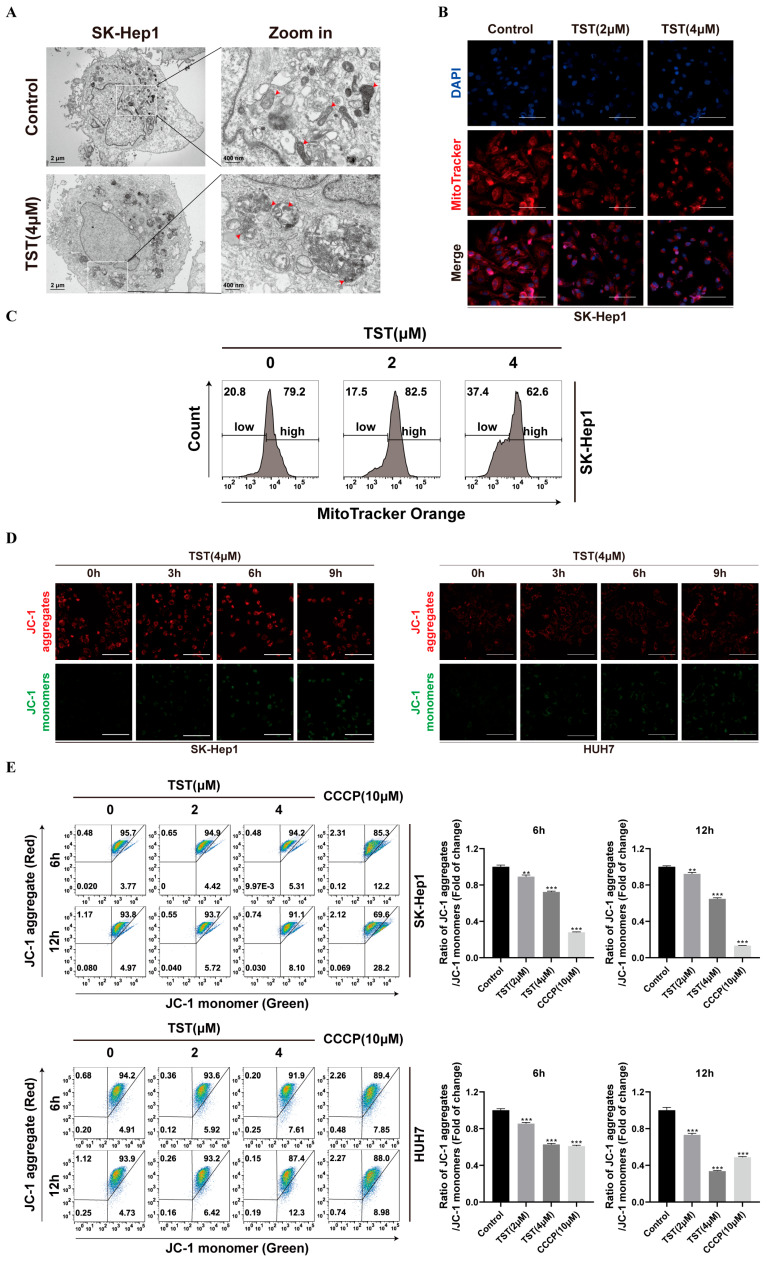
TST induces mitochondrial impairment in HCC cells. (**A**) Mitochondrial structure in TST-treated SK-Hep1 cells under electron microscope. The red arrows indicate mitochondria. (**B**) SK-Hep1 cells treated with TST for 24 h were stained with MitoTracker^®^ Orange CMTMRos and subsequently observed under a fluorescence microscope. (**C**) The percentage of cells exhibiting low MitoTracker fluorescence was quantified using flow cytometry. (**D**) HCC cells treated with TST (4 μM) for the indicated time point were stained with JC-1 dye and subsequently observed under a fluorescence microscope. Red fluorescence signifies the accumulation of JC-1 in normal mitochondria, while green fluorescence indicates the presence of JC-1 monomers in the cellular matrix due to the reduction in MMP. (**E**) The MMP of SK-Hep1 and HUH7 cells treated with TST or the mitochondrial uncoupler CCCP for the indicated time point was assessed with flow cytometry. Changes in MMP are shown in histograms. Scale bar: 100 μm. ** *p* < 0.01, and *** *p* < 0.001 versus the control group. CCCP: carbonyl cyanide m-chlorophenyl hydrazone.

The cellular phenotypic changes of subcellular organelles were observed with transmission electron microscopy (TEM) upon TST exposure. Notably, the mitochondria in TST-treated cells exhibited obvious damage compared to those in the control group through TEM observations (Figure 4A and Figure S2A). Furthermore, the mitochondrial fluorescent probe MitoTracker^®^ Orange was used to label functionally normal mitochondria. As shown in Figure 4B, TST caused a decrease in fluorescence in SK-Hep1 cells, as well as in HUH7 cells (Figure S2B), indicating a reduction in the number of healthy mitochondria. Flow cytometry analysis also demonstrated a significant increase in the percentage of cells with low MitoTracker fluorescence under TST treatment (Figure 4C and Figure S2C), suggesting that TST may impair mitochondrial function in HCC cells. Changes in the MMP were further detected. With the prolongation of TST treatment, the red fluorescence (JC-1 aggregates) representing intact mitochondria weakened, while the green fluorescence (JC-1 monomers) representing depolarized mitochondria gradually increased (Figure 4D). Additionally, the ratio of red to green fluorescence also decreased, indicating that TST treatment indeed lowered the MMP in HCC cells (Figure 4E). In summary, these findings illustrated that TST caused mitochondrial impairment in HCC cells.

### 2.5. Mitochondrial Damage in HCC Cells Triggered by TST Relies on ROS

**Figure 5:**
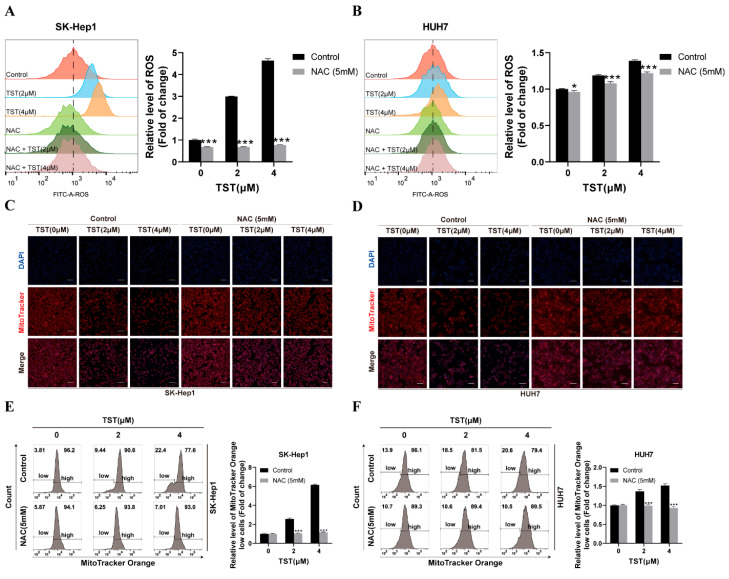
TST-induced mitochondrial damage in HCC cells depends on ROS. (**A**,**B**) ROS in HCC cells treated with TST in the absence or presence of NAC for 24 h were assessed with flow cytometry. * *p* < 0.05, and *** *p* < 0.001 versus the control group. (**C**,**D**) HCC cells treated with TST in the absence or presence of NAC for 24 h were stained with MitoTracker^®^ Orange CMTMRos and subsequently observed under a fluorescence microscope. (**E**,**F**) Quantification of the fluorescence intensity in the population of cells exhibiting low MitoTracker fluorescence by flow cytometry. Scale bar: 100 μm. NAC: N-acetyl-L-cysteine.

Given that ROS are recognized as a vital factor that cause damage to mitochondria [23], we explored the induction of ROS by TST in HCC cells. The ROS level stained with 2,7-dihydrodichlorofluorescein diacetate was measured using flow cytometry and was found to increase clearly under TST treatment (Figure S3). With the addition of NAC, an ROS scavenger, the ROS induced by TST were inhibited, as shown in Figure 5A,B, and the number of healthy mitochondria in TST-treated HCC cells, which were labeled with MitoTracker^®^ Orange CMTMRos, was restored as well (Figure 5C–F). These findings suggested that TST caused mitochondrial impairment in HCC cells was mediated by ROS induction.

### 2.6. TST Suppresses the Growth of HCC Cells in an ROS-Dependent Manner

**Figure 6:**
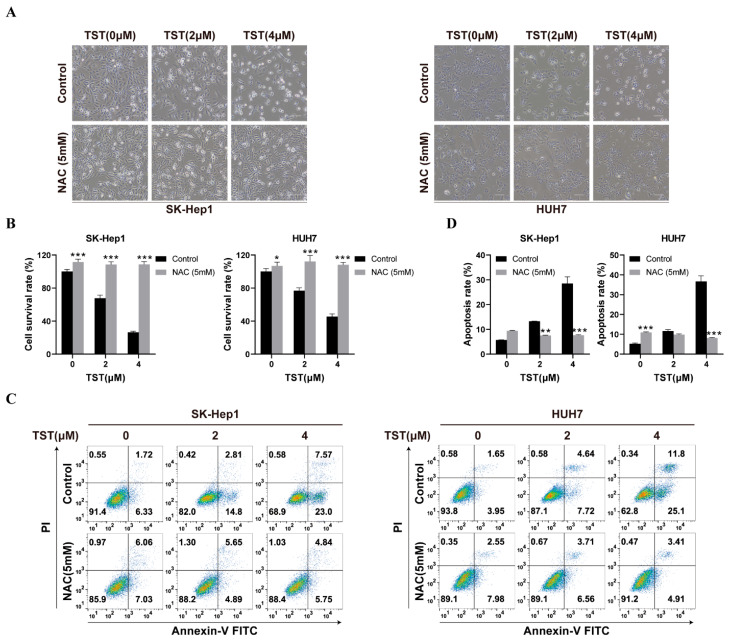
ROS scavenging with NAC eliminates the inhibitory activity of TST against HCC cells. (**A**) Graphic representation of cell morphology after treatment with TST in the absence or presence of NAC (5 mM) for 24 h. (**B**) The cellular viability of HCC cells treated with TST in the absence or presence of NAC (5 mM) for 24 h was assessed using MTS solution. * *p* < 0.05, ** *p* < 0.01, and *** *p* < 0.001 versus the control group. (**C**) Apoptosis of HCC cells treated with TST in the absence or presence of NAC (5 mM) for 24 h was assessed with flow cytometry. (**D**) Statistical analysis of cell apoptosis rate. Scale bar: 100 μm.

Since TST-induced mitochondrial damage relied on ROS induction, we next investigated whether the inhibitory activity of TST on HCC cells was ROS-dependent. TST treatment decreased the amount of SK-Hep1 and HUH7 cells and caused them to shrink (Figure 6A), whereas pretreatment with the ROS scavenger NAC entirely reversed this effect and eliminated the cytotoxicity of TST against HCC cells (Figure 6B). Furthermore, cell apoptosis analysis was performed with flow cytometry. The treatment of NAC consistently suppressed the apoptosis induced by TST in HCC cells (Figure 6C,D), indicating ROS-mediated mitochondrial impairment plays an indispensable role in the TST-induced apoptosis of HCC cells

### 2.7. Mitophagy Blockade Enhances the Inhibitory Activity of TST against HCC Cells

**Figure 7:**
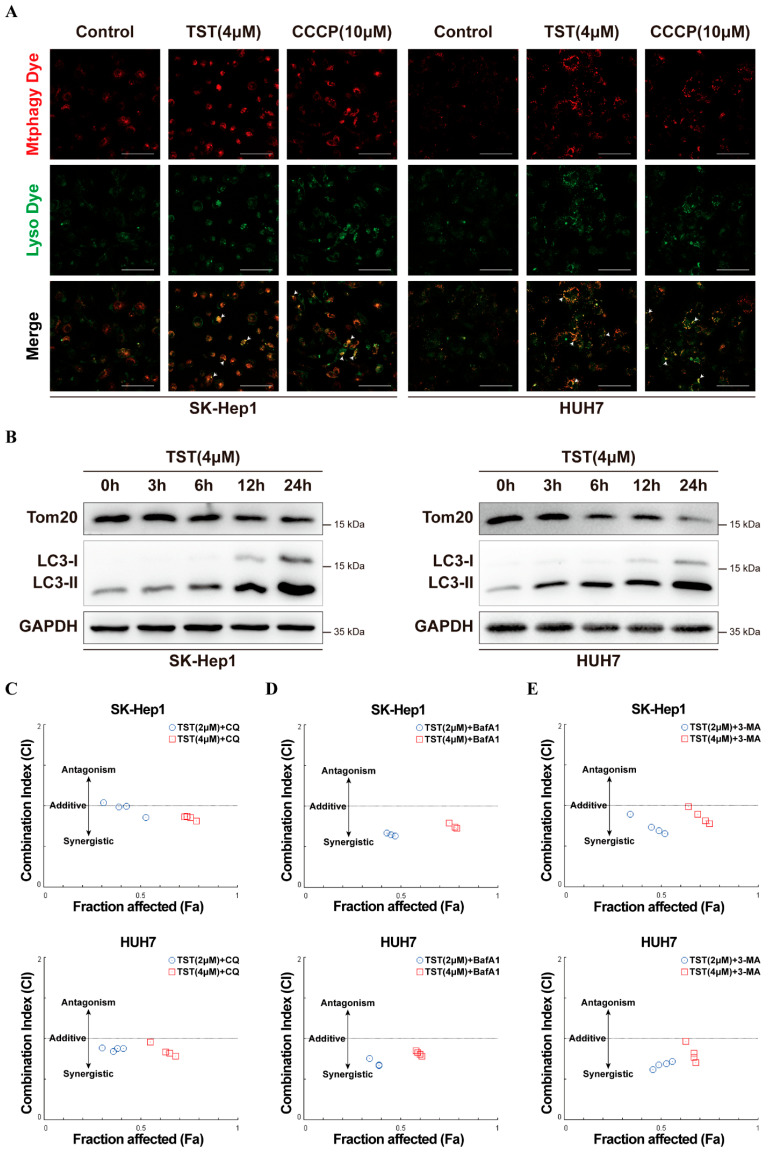
Mitophagy blockade enhances the inhibitory activity of TST against HCC cells. (**A**) HCC cells treated with TST or CCCP for 12 h were stained with Mtphagy dye and Lyso dye, with mitophagy observed under a fluorescence microscope. The arrows show the co-localization of Mtphagy dye and Lyso dye, indicating the presence of mitophagy. (**B**) Western blot analysis revealed the protein levels of Tom20 and LC3I/II in HCC cells treated with TST (4 μM) for the indicated time point. Graphic representation of CI plot (Fa-CI plot) obtained from the CompuSyn software (version 1.0): (**C**) TST (2 μM, 4 μM) in combination with CQ (2, 4, 6, and 8 μM) for 24 h; (**D**) TST (2 μM, 4 μM) in combination with BafA1 (100, 200, 400, and 800 nM) for 24 h; (**E**) TST (2 μM, 4 μM) in combination with 3-MA (2, 4, 6, and 8 mM) for 24 h. CI < 1 indicates synergistic, CI = 1 indicates additive, and CI > 1 indicates antagonistic. 3-MA, 3-methyladenine; BafA1, bafilomycin A1; CQ, chloroquine.

Increasing mitophagy to remove damaged mitochondria and maintain cellular function was frequently reported in response to mitochondria impairment, which may result in drug resistance. We subsequently investigated whether mitophagy happens in TST-treated HCC cells. To visualize mitophagy, we used Mtphagy, which specifically binds to mitochondria and shifts from weak to strong red fluorescence when mitochondria are in a lysosomal acidic environment. In parallel, Lyso was employed to label lysosomes, presenting as green fluorescence. As shown in Figure 7A, the mitochondrial uncoupling agent carbonyl cyanide m-chlorophenyl hydrazone (CCCP), as a positive control, caused an increase in yellow fluorescence spots (indicative of the co-localization of red and green fluorescence). A more pronounced increase in the number of yellow fluorescence spots was also observed upon TST treatment, suggesting that TST induces mitophagy in HCC cells. The mitochondrial outer membrane protein Tom20 is commonly used to monitor mitochondrial numbers during mitophagy, and a decrease in Tom20 expression is considered indicative of reduced mitochondrial numbers and upregulated mitophagy. Western blot analysis unveiled that TST induced a time-dependent decrease in the mitophagy marker Tom20 and a time-dependent accumulation of the autophagic marker protein LC3II in HCC cells (Figure 7B).

We next investigated the role of mitophagy in TST-induced inhibition of HCC cell growth. In SK-Hep1 and HUH7 cells, LC3 was silenced using siRNA, followed by treatment with TST. As shown in Figure S4A, the mitophagy was inhibited in LC3 siRNA-targeted HCC cells. On the contrary, the cytotoxicity and TST-induced cell apoptosis of HCC cells were effectively enhanced (Figure S4B–D), which indicated that mitophagy here resulted in the resistance of HCC cells to TST treatment.

Mitophagy inhibitors chloroquine (CQ), bafilomycin A1 (BafA1), and 3-methyladenine (3-MA) were then individually employed in combination with TST to treat HCCs, and the combination index (CI) of cytotoxicity was calculated and plotted. As illustrated in Figure 7C–E, treatment with TST in combination with mitophagy inhibitors exhibited synergistic inhibitory activity against HCC cells (CI < 1). These results suggested that the mitophagy conferred HCC cells resistance to TST treatment, and the mitophagy blockade efficiently enhanced the inhibitory effect of TST on HCC cells.

## 3. Discussion and Conclusions

Given the poor efficacy and diversity of current anti-liver cancer drugs, there is an urgent need for novel therapeutic developments. In this context, the drug repurposing strategy emerges as a vital approach in drug development, especially in the realm of anti-tumor drugs. With advancements in bioinformatics, this strategy is increasingly being employed and holds promise for the discovery of innovative liver cancer treatments.

In the study, we utilized a comprehensive approach integrating computational and experimental techniques and identified the antibacterial drug TST as a promising anti-liver cancer agent for the first time using the PRISM drug database. Our findings demonstrated that TST halted the cell cycle, triggered cell apoptosis, and impeded the proliferation of HCC cells. TST also possessed the capability to suppress cell migration. Further research found that TST triggered apoptosis of HCC cells through ROS-mediated mitochondrial impairment. Additionally, TST induced mitophagy, and the blockade of mitophagy could effectively enhance the anti-liver cancer activity of TST.

TST was reported to target FOXM1 and inhibit tumor growth in cases of osteosarcoma [19], acute lymphoblastic leukemia [24], gastric cancer [20], and breast cancer [21,25]. TST binds to FOXM1 and thereby reduces its expression to promote an increase in ROS, subsequently regulating the expression of apoptosis-related proteins to trigger apoptosis. Whether FOXM1 is involved in TST-induced apoptosis of HCC is worthy of further study. In malignant mesothelioma, PRDX3, a mitochondrial protein, was reported as the target of TST, leading to disruption of antioxidant capacity and the subsequent release of ROS [26]. The upregulation of PRDX3 was detected in over 90% of liver cancer patients [27], suggesting that PRDX3 may be also the potential target for TST in HCC cells, which deserves further investigation.

It has been reported that TST induces mitophagy in macrophages [28]. In this study, for the first time, we found that TST induced mitophagy in tumor cells. Mitophagy, the selective autophagy of mitochondria [14], has emerged as a prominent area of research that has garnered extensive attention in relation to clinical diseases such as neurodegenerative diseases, metabolic diseases, and cancer [29]. The current research demonstrated that mitophagy helped with the removal of damaged mitochondria induced by TST, resulting in drug-resistant HCC cells. Blocking mitophagy with LC3 knockdown or autophagy inhibitors CQ, BafA1, and 3-MA efficiently enhanced the anti-tumor activity of TST. In some cancers, such as gastric cancer and osteosarcoma, TST induces autophagy to promote tumor cell apoptosis and inhibit cell proliferation [20,30], suggesting that autophagy plays different roles in TST-induced cell apoptosis according to different cellular contexts.

TST is currently undergoing clinical trials for the treatment of mesothelioma in the United Kingdom (ClinicalTrials.gov identifier: NCT05278975), indicating it a promising candidate for an anti-liver cancer agent. In the present study, we experimentally investigated the anti-liver cancer activity of TST and elucidated that TST induces cell apoptosis through ROS-mediated mitochondrial damage, offering valuable data for the further development of TST as an anti-HCC agent.

## 4. Material and Methods

### 4.1. PRISM Dataset and Drug Screening

The PRISM repurposing dataset, a publicly available database comprising information on the inhibitory activity of 4518 drugs against 578 human cancer cell lines, was established for the purpose of investigating non-oncology drugs for potential use in tumor treatment. The drug screening dataset is segmented into primary screening and secondary screening data. In the secondary screening, 1448 drugs selected from the primary screening were further evaluated for their activity against 499 human tumor cell lines at doses of 610 pM, 2.4 nM, 9.8 nM, 39 nM, 156 nM, 625 nM, 2.5 μM, and 10 μM.

We only extracted anti-cancer activity data for non-tumor drugs, which accounted for 53% of the pool of 1448 drugs. Subsequently, the cell viability inhibition data for drugs at a dose of 625 nM were analyzed, and drugs causing over 70% cell viability inhibition in at least 80% of the tested cancer cell lines were selected, as instructed [22]. Inexpensive and easily available drugs that have undergone at least phase I clinical studies and have no reported activities against liver cancer were finally selected for further investigation in the present study.

### 4.2. Reagent and Plasmids

Dulbecco’s modified Eagle’s medium (DMEM, C3101-0500), fetal bovine serum (FBS, C2910-0500), and penicillin–streptomycin solution (C3421-0100) were bought from VivaCell (Shanghai, China). TST (purity > 99%, HY-B0990) was purchased from MedChemExpress (Monmouth Junction, NJ, USA). CCCP (S6494), 3-MA (S2767), BafA1 (S1413), and CQ (S6999) were bought from Selleck (Houston, TX, USA). NAC (A9165) was obtained from Sigma-Aldrich (St. Louis, MO, USA). Lipofectamine^®^ 3000 reagent (L3000015) was obtained from Invitrogen (Camarillo, CA, USA). All antibodies used in this study are listed in Supplementary Table S1.

### 4.3. Cell Culture

The human HCC cell lines (Hep3B, HUH7, SK-Hep1, HepG2, HCCLM3, PLC/PRF/5) were obtained from the Shanghai Institute of Biochemistry and Cell Biology, Chinese Academy of Sciences (Shanghai, China). All cells were cultured following the guidelines.

### 4.4. Cell Viability and Colony Formation Assay

CellTiter 96^®^ Aqueous One Solution Reagent (G3581, Promega; Madison, WI, USA) was utilized to evaluate the effects of TST on cell viability. A total of 4000 cells per well were plated in 96-well plates, then exposed to TST for 24 h and 48 h. The subsequent experimental procedures were conducted as previously described [31].

For colony formation assays, 1000 cells per well were seeded in 6-well plates and incubated in a medium containing TST for 1–2 weeks. Once colony formation became visible to the naked eye, the experiment proceeded as previously described [31].

### 4.5. Drug Combination Analysis

To assess the drug interactions between the mitophagy inhibitors chloroquine (CQ), bafilomycin A1 (BafA1), and 3-methyladenine (3-MA) and TST, analysis was conducted using CompuSyn software (version 1.0) based on the Chou-Talalay method [32]. The two drugs were used in combination at non-equimolar ratios, with TST concentrations selected at 2 and 4 μM and mitophagy inhibitor concentrations as follows: CQ (2, 4, 6, and 8 μM), BafA1 (100, 200, 400, and 800 nM), and 3-MA (2, 4, 6, and 8 mM). Liver cancer cells were treated with these drugs individually or in combination for 24 h, followed by cell viability assessment using the MTS assay. To evaluate the synergistic effect of two drugs, the cell mortality rate of the individual drug and the corresponding combined treatment were input into the CompuSyn software (version 1.0), which uses the Chou-Talalay method to calculate the CI and generate CI plots. In these plots, the *x*-axis represents the fraction affected (Fa), and the *y*-axis represents the CI, facilitating the assessment of drug synergism between the drug combinations. CI < 1 indicates a synergistic effect, CI = 1 indicates an additive effect, and CI > 1 indicates an antagonistic effect.

### 4.6. Cell Cycle Analysis

A total of 2 × 10^5^ cells per well were planted in 6-well plates, followed by the treatment of attached cells with TST for 24 h. The cell cycles were subsequently analyzed as previously described [33].

### 4.7. Apoptosis Assay

Cell apoptosis was assessed using the Annexin V-FITC/PI apoptosis detection kit (FXP018-100, 4A Biotech; Beijing, China) following the manufacturer’s instructions. In brief, 1 × 10^5^ cells adhered to a 6-well plate were subjected to TST for 24 h or 48 h. After treatment, cells underwent cell apoptosis analysis as previously described [34], and the fluorescence intensity was measured using the FACSCalibur flow cytometer (FACSCelesta, BD Biosciences; East Rutherford, NJ, USA).

### 4.8. Western Blot Assay

Cells treated with compounds or transfected with siRNA were collected and the total protein was extracted using a strong RIPA buffer (P0013B, Beyotime; Shanghai, China) supplemented with PMSF (7110-OP, Sigma-Aldrich) and a phosphatase inhibitor cocktail (04906845001, Roche; Madison, WI, USA). The resulting supernatants were then quantified with a BCA kit (P0009, Beyotime). Equal amounts of each protein extract were then separated by SDS-PAGE and transferred onto PVDF membranes (ISEQ00010, Millipore; Billerica, MA, USA). After blocking, the membranes were incubated overnight at 4 °C with specific primary antibodies. The following day, the membranes were incubated with appropriate secondary antibodies at room temperature for 1 h. Finally, the target protein bands were visualized using an ImageQuant LAS 4000 mini (GE Healthcare, Chicago, IL, USA) after exposure to the ECL substrate (32106, Thermo Fisher; Waltham, MA, USA). Details of the primary and secondary antibodies used can be found in Supplementary Table S1.

### 4.9. Immunofluorescence Assay

Cells (2 × 10^5^ cells/well) seeded in 12-well plates were treated with various concentrations of TST for 12 h. Following treatment, the subsequent fluorescence experiments were performed as previously described [31].

For the immunofluorescence assay involving fluorescent dyes, we used the MitoTracker^®^ Orange CMTMRos (M7510, Thermo Fisher; Waltham, MA, USA), a mitochondrial membrane potential assay kit with JC-1 (C2006, Beyotime; Shanghai, China), and a mitophagy detection kit (MD01, DOJINDO; Kumamoto, Japan).

JC-1 is a fluorescent probe widely used for detecting MMP. Its mechanism operates as follows: when the MMP is normal, JC-1 accumulates in the mitochondrial matrix, forming J-aggregates that emit red fluorescence. However, when the membrane potential is low, JC-1 remains in its monomeric form, emitting green fluorescence.

The mitophagy detection kit includes two dyes: Mtphagy dye and Lyso dye. Mtphagy dye is designed to localize to mitochondria within the cell, exhibiting weak fluorescence under normal conditions. During mitophagy, when damaged mitochondria fuse with lysosomes to form an acidic environment, the Mtphagy dye fluoresces more strongly. Lyso dye, on the other hand, labels lysosomes and emits green fluorescence.

Cells were seeded and the staining procedure was performed following the instructions provided for each dye, and the cells were then observed under a fluorescence microscope (Eclipse, Nikon, Japan).

### 4.10. RNA Interference

RNA interference was carried out by transfecting cells with RNA oligos using Lipofectamine^®^ 3000 (L3000015, Invitrogen; Camarillo, CA, USA) for 48 h, and the sequences of the RNA oligos are listed in Supplementary Table S2.

### 4.11. Cell Scratch Assay

A total of 2 × 10^5^ cells per well were planted in 12-well plates and grown to at least 90% confluence. Scratches were made, followed by washing of the cells with PBS. Afterwards, the cells were incubated in a medium with 1% serum and TST. Photographs were taken immediately after scratching and again 12 h or 24 h later. The scratch assay was quantified using ImageJ software (version 1.53) to calculate the scratch distances. The migration rate was determined as follows: the migration distance for each group was calculated by subtracting the final scratch distance (at 12 h or 24 h) from the initial distance (0 h). The obtained value for each group was then expressed as a percentage of the migration distance of the untreated control cells.

### 4.12. Determination of ROS

Cells were treated with TST, harvested into clean centrifuge tubes, and stained with a fluorescent dye using a reactive oxygen species assay kit (S0033S, Beyotime) as per the manufacturer’s instructions. Then, the cells were washed with phosphate-buffered saline (PBS), and the fluorescence intensity of each group was detected using the FACSCalibur flow cytometer (FACSCelesta, BD Biosciences; East Rutherford, NJ, USA).

### 4.13. Determination of Mitochondrial Mass

Cells were treated with TST and stained with MitoTracker^®^ Orange CMTMRos (M7510, Thermo Fisher; Waltham, MA, USA) following the instructions provided. Subsequently, a FACSCalibur flow cytometer (FACSCelesta, BD Biosciences; East Rutherford, NJ, USA) was used to evaluate the overall mitochondrial mass in each group.

### 4.14. MMP Measurement

Cells treated with TST were collected and stained with JC-1 dye using the mitochondrial membrane potential assay kit (C2006, Beyotime; Shanghai, China) according to the instructions provided. Afterwards, the cells were washed with JC-1 buffer and the fluorescence intensity of each group was measured using the FACSCalibur flow cytometer (FACSCelesta, BD Biosciences; East Rutherford, NJ, USA). The ratio of aggregates (red fluorescence) to monomers (green fluorescence) served as an indicator of the extent of damage to the MMP.

### 4.15. TEM Assay

Cells were treated with TST for 12 h, then washed with PBS and incubated with an electron microscope fixative. Subsequent experimental procedures were performed as previously described [35].

### 4.16. Statistical Analysis

Statistical analysis of the data was performed using two-tailed Student’s *t*-tests and two-way ANOVA, employing the GraphPad Prism software (version 8.0) for data analysis. Significance levels were denoted as follows: * *p* < 0.05, ** *p* < 0.01, and *** *p* < 0.001. *p*-values below 0.05 (*p* < 0.05) were deemed statistically significant.

## Data Availability

Data are available upon reasonable request.

## Supplementary Materials

The following supporting information can be downloaded at: https://www.mdpi.com/article/10.3390/ijms25179717/s1.
