# Supplementary material for: Thiostrepton as a Potential Therapeutic Agent for Hepatocellular Carcinoma
Source: Int J Mol Sci. 2024 Sep 8;25(17):9717. doi: 10.3390/ijms25179717 (PMC11395809; doi:10.3390/ijms25179717)
Supplement: Supplementary file 1 [file ijms-25-09717-s001.zip › ijms-3156132-supplementary.pdf]

Supplementary Material

Content:

Supplementary methods ……………………………………………………………p.2

Table S1. Antibodies …………………………………………………………… p.3

Table S2. RNA oligos used for RNA interference……………………………… p.3

Figure S1…………………………………………………………………………p.4

Figure S2…………………………………………………………………………p.6

Figure S3…………………………………………………………………………p.7

Figure S4…………………………………………………………………………p.8

Supplementary methods

1. Drug repurposing assisted by bioinformatics

The PRISM repurposing dataset, a publicly available database comprising information on the inhibitory activity of 4518 drugs against 578 human cancer cell lines, was established for the purpose of investigating non-oncology drugs for potential use in tumor treatment. The drug screening dataset is segmented into primary screening and secondary screening data. In the secondary screening phase, 1448 drugs selected from the primary screening were further evaluated for their activity against 499 human tumor cell lines at doses of 610 pM, 2.4 nM, 9.8 nM, 39 nM, 156 nM, 625 nM, 2.5 μM, and 10 μM.

We extracted anti-cancer activity data of only non-tumor drugs, which accounted for 53% of the pool of 1448 drugs. Subsequently, cell viability inhibition data of drugs at the dose of 625 nM were analyzed and drugs causing over 70% cell viability inhibition in at least 80% of the tested cancer cell lines were selected, as instructed[22]. Inexpensive and easily available drugs that have undergone at least phase I clinical studies and have no reported activities against liver cancer were finally selected for further investigation in the present study.

Supplementary Table S1. Antibodies for Western blotting and immunofluorescence.

Antigen	Species	Applications and dilutions	Source
GAPDH	Mouse	WB (1:50000)	Proteintech #60004-1-Ig
N-cadherin	Rabbit	WB (1:2000)	Proteintech #22018-1-AP
SNAI1	Mouse	WB (1:500)	Santa Cruz #sc-271977
SNAI2/SLUG	Rabbit	WB (1:5000)	Proteintech #12129-1-AP
Vimentin	Rabbit	WB (1:2000)	Proteintech #10366-1-AP
Beta Actin	Mouse	WB (1:20000)	Proteintech #66009-1-Ig
PARP	Rabbit	WB (1:1000)	Cell Signaling Technology #9542
Caspase 3	Rabbit	WB (1:1000)	Proteintech #19677-1-AP
Caspase 9	Rabbit	WB (1:1000)	Proteintech #10380-1-AP
Bcl-2	Rabbit	WB (1:1000)	Proteintech #12789-1-AP
FOXM1	Mouse	WB (1:500)	Santa Cruz #sc-376471
LC3B	Rabbit	WB (1:1000)	Cell Signaling Technology #3868
Tom20	Mouse	WB (1:1000)	Cell Signaling Technology #13929
**Secondary goat anti-rabbit antibody**	goat	WB (1:10000)	Invitrogen #A24537
**Secondary goat-anti mouse antibody**	goat	WB (1:10000)	Invitrogen #A24524

Cell Signaling Technology, Boston, MA, USA; Invitrogen, Carlsbad, CA, USA; Proteintech, Wuhan, China; Santa Cruz, Santa Cruz, CA, USA.

Supplementary Table S2. RNA oligos used for RNA interference.

Name	Target sequence	Supplier
Negative control siRNA	UUCUCCGAACGUGUCACGUTT	TsingKe
siLC3	GAGUGAGAAAGAUGAAGAU	TsingKe

Tsingke, Beijing, China.

Supplementary Figure S1

A

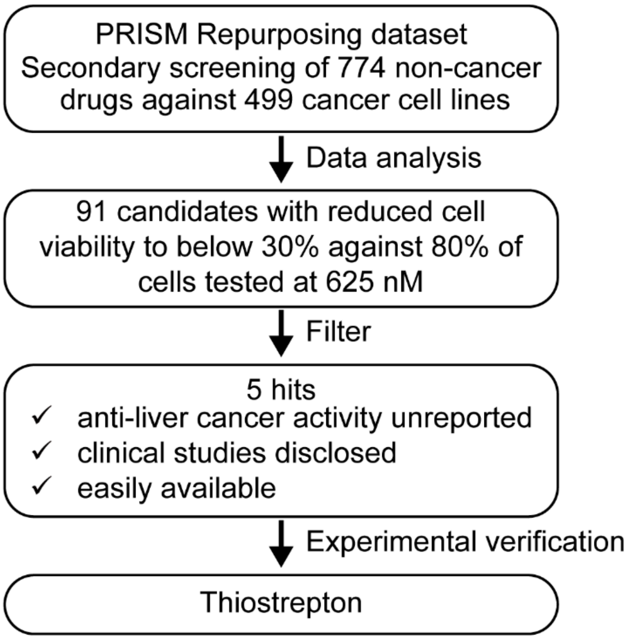

B

Drug	percentage	Drug	percentage
oligomycin-a	99.79%	clonazepam	88.36%
digoxin	99.77%	D-7193	88.22%
colchicine	99.74%	fenbendazole	87.89%
monensin	99.70%	narasin	87.85%
pyrithione-zinc	99.57%	broxyquinoline	87.69%
EVP4593	99.57%	monensin	87.63%
brilliant-green	99.55%	asymmetrical-dimethylarginine	87.42%
ONX-0914	99.13%	difluprednate	87.02%
sirolimus	99.13%	deoxycorticosterone-acetate	85.50%
aphidicolin	98.51%	terbutaline	85.23%
nanchangmycin	98.50%	CGS-15943	85.04%
crystal-violet	98.46%	benzethonium	84.60%
disulfiram	98.29%	FPH1-(BRD-6125)	84.58%
radezolid	97.98%	PSI-7976	84.32%
narasin	97.64%	tanaproget	84.01%
anisomycin	96.78%	nonoxynol-9	83.80%
tedizolid	96.77%	latrepirdine	83.56%
adaprev	96.72%	FK-3311	83.51%
thiram	96.64%	BI-78D3	83.45%
rutin	96.41%	azalomycin-b	83.41%
valnemulin	96.35%	LE-135	83.23%
SR-33805	96.27%	merimepodib	83.22%
ivermectin	96.19%	mexiletine	83.04%
tedizolid-phosphate	95.77%	mebendazole	82.84%
thiostrepton	95.50%	adefovir-dipivoxil	82.51%
penfluridol	94.97%	norepinephrine	82.42%
flubendazole	94.48%	LDN-212854	82.23%
dicycloverine	94.47%	diazooxonorleucine	81.99%
brivaracetam	94.23%	tepoxalin	81.74%
disulfiram	93.95%	mycophenolic-acid	81.66%
pirenperone	93.84%	fluorometholone	81.43%
albendazole	93.50%	VX-765	81.21%
SB-218078	93.05%	maxacalcitol	81.06%
puromycin	92.87%	papaverine	80.99%
lanatoside-c	92.46%	triclabendazole	80.95%
mefexamide	92.14%	RN-1734	80.86%
mozavaptan	91.90%	paricalcitol	80.82%
salinomycin	91.87%	JIB04	80.82%
CHIR-124	91.69%	paliperidone	80.72%
JTE-607	91.68%	oxyphencyclimine	80.51%
digitoxigenin	91.58%	drospirenone	80.47%
cetylpyridinium	90.36%	broxaldine	80.21%
spiradoline	89.98%	fendiline	80.17%
Ro-106-9920	89.07%	florfenicol	80.04%
pardoprunox	89.01%	ABT-702	80.00%
darifenacin	88.89%		

C

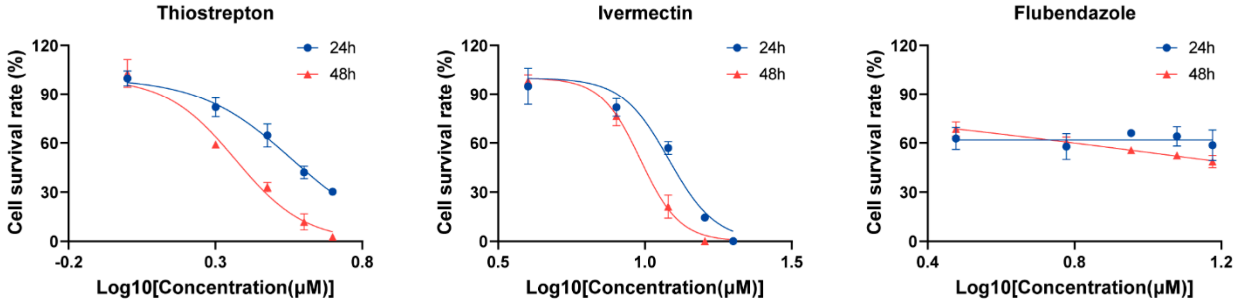

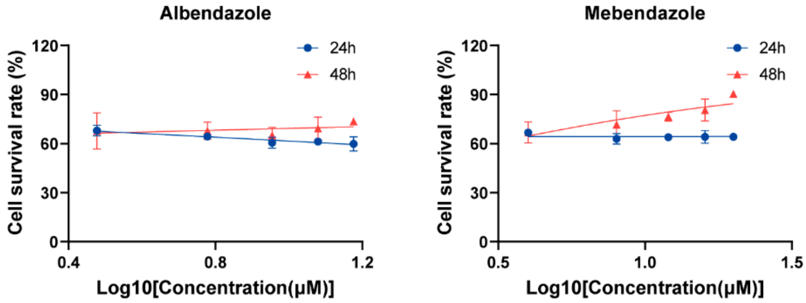

Non-oncology compounds	IC_{50}(μM)	
	24h	48h
Thiostrepton	3.64	2.33
Ivermectin	12.05	9.65
Flubendazole	>15	14
Albendazole	>15	>15
Mebendazole	>15	>15

Figure S1. Drug screening assisted by bioinformatics. (A) The graphical presentation of the virtual drug screening and experimental verification process. First, we obtained the drug secondary screening data from the PRISM database, which

include the inhibitory activity of 774 non-cancer drugs on 499 cancer cell lines. Subsequently, to narrow down the range of drug candidates, we selected drugs that, at a concentration of 625 nM, reduced cell viability to below 30%, in at least 80% of the total cell lines tested, and obtained 91 candidate drugs with broad-spectrum anticancer activity. Next, we narrowed down the drug candidates to five, including thiostrepton, ivermectin, flubendazole, albendazole, and mebendazole, based on further criteria: having undergone at least phase I clinical studies, having no reports of anti-liver cancer activity, and being easily available. We further tested the activity against liver cancer cells, and TST exhibited the most potent anti-liver cancer activity. (B) The 91 non-tumor drugs and their percentage inhibition on tumor cells. (C) The cellular viability of SK-Hep1 cells incubated with various concentrations of thiostrepton, ivermectin, flubendazole, albendazole, and mebendazole for 24 and 48 h was assessed using MTS solution. The IC50 for each drug was calculated based on its dose–response curve.

Supplementary Figure S2

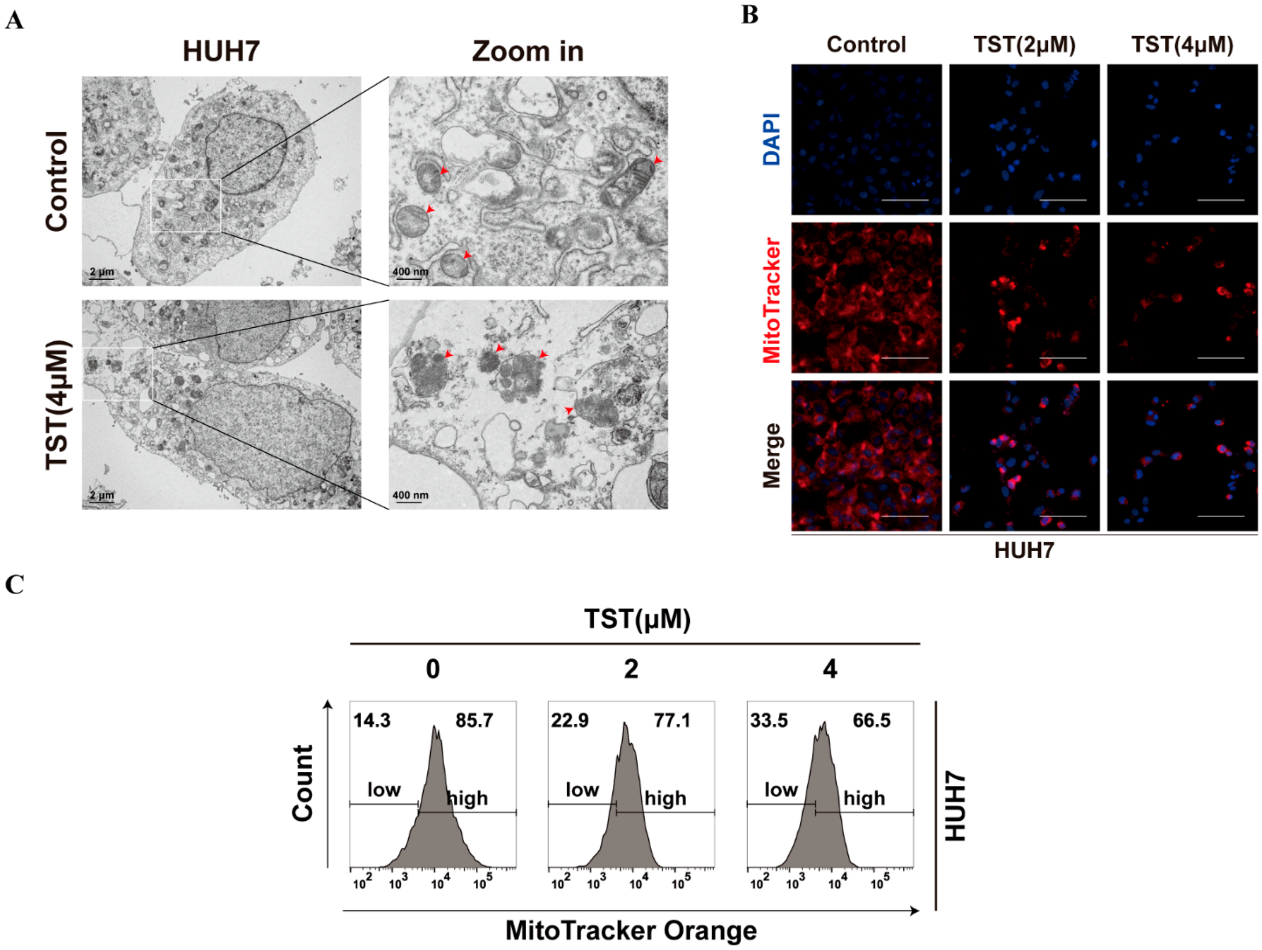

Figure S2. TST induces mitochondrial impairment in HCC cells. (A) Mitochondrial structure in TST-treated HUH7 cells under electron microscope. The red arrows indicate mitochondria. (B) HUH7 cells treated with TST for 24 h were stained with MitoTracker® Orange CMTMRos and subsequently observed under a fluorescence microscope. (C) The percentage of cells exhibiting low MitoTracker fluorescence was quantified using flow cytometry. Scale bar: 100 μm. *p< 0.05, **p< 0.01, and ***p < 0.001 versus the control group.

Supplementary Figure S3

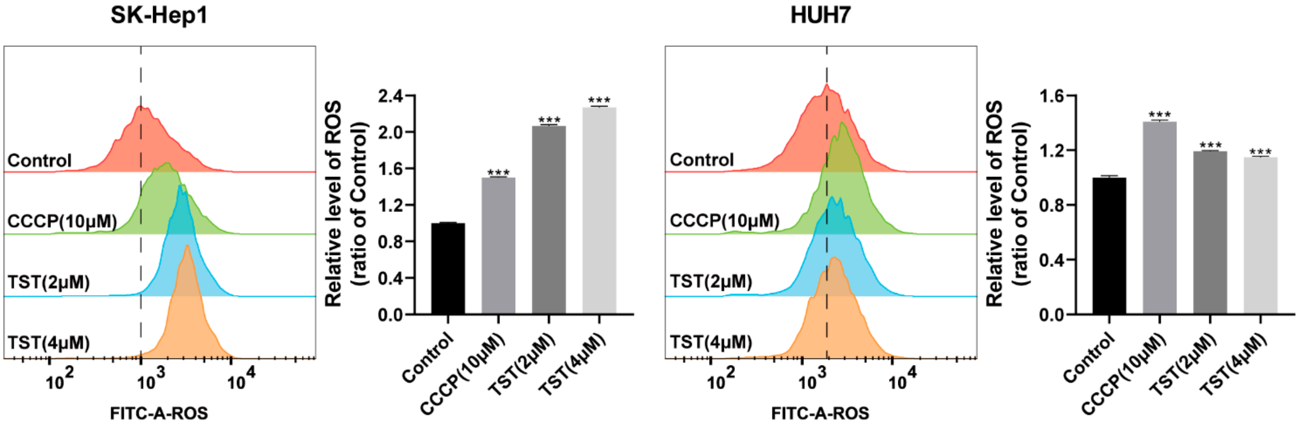

Figure S3. TST induces ROS production in HCC cells. ROS of HCC cells treated with TST or CCCP for 12 h were assessed by flow cytometry. *p< 0.05, **p< 0.01, and ***p < 0.001 versus the control group.

Supplementary Figure S4

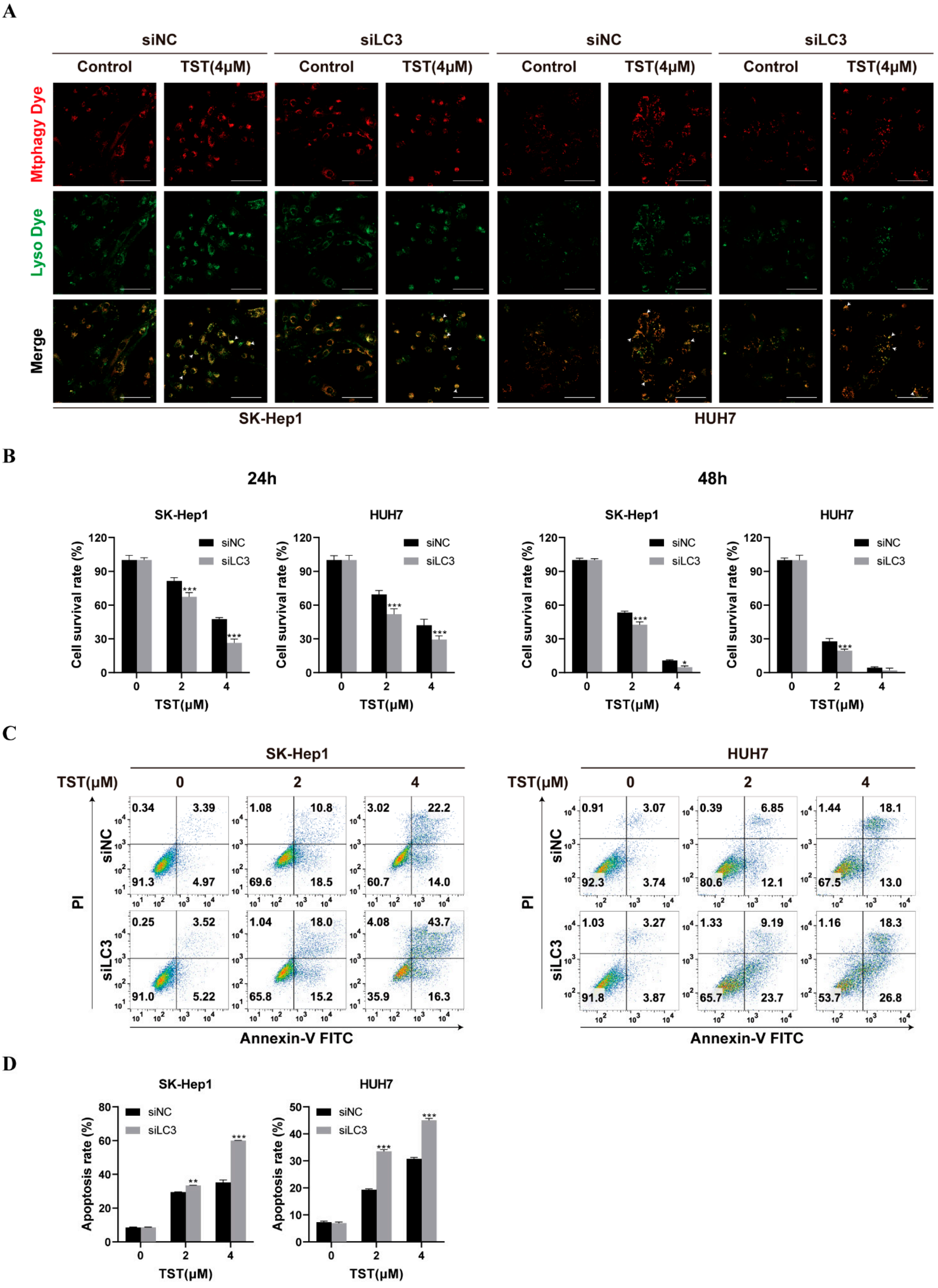

Figure S4. Mitophagy blockade enhances the anti-liver cancer activity of TST. (A) HCC cells transfected with negative control or LC3 siRNA were treated with TST for 12 h, mitophagy was observed under a fluorescence microscope using Mtphagy dye and Lyso dye staining. The arrows show the co-localization of MitoPhagy dye and Lyso dye, indicating the presence of mitochondrial autophagy. (B) The cellular viability of HCC cells transiently transfected with negative control or LC3 siRNA for 48 h, followed by exposure to TST for 24 h or 48 h, was assessed using MTS solution. *$p< 0.05$, **$p< 0.01$, and ***$p < 0.001$ versus the control group. (C) Apoptosis of HCC cells transiently transfected with negative control or LC3 siRNA for 48 h, followed by treatment with TST for 24 h, was assessed by flow cytometry. (D) Statistical analysis of cell apoptosis rate. *$p< 0.05$, **$p< 0.01$, and ***$p < 0.001$ versus the control group.
